# Induction of Trained Immunity by Recombinant Vaccines

**DOI:** 10.3389/fimmu.2020.611946

**Published:** 2021-01-07

**Authors:** Camila Covián, Mariana Ríos, Roslye V. Berríos-Rojas, Susan M. Bueno, Alexis M. Kalergis

**Affiliations:** ^1^ Millenium Institute on Immunology and Immunotherapy, Departamento de Genética Molecular y Microbiología, Facultad de Ciencias Biológicas, Pontificia Universidad Católica de Chile, Santiago, Chile; ^2^ Departamento de Endocrinología, Facultad de Medicina, Escuela de Medicina, Pontificia Universidad Católica de Chile, Santiago, Chile

**Keywords:** recombinant BCG, trained immunity, unspecific cross-protection, respiratory syncytial virus, metapneumovirus

## Abstract

Vaccines represent an important strategy to protect humans against a wide variety of pathogens and have even led to eradicating some diseases. Although every vaccine is developed to induce specific protection for a particular pathogen, some vaccine formulations can also promote trained immunity, which is a non-specific memory-like feature developed by the innate immune system. It is thought that trained immunity can protect against a wide variety of pathogens other than those contained in the vaccine formulation. The non-specific memory of the trained immunity-based vaccines (TIbV) seems beneficial for the immunized individual, as it may represent a powerful strategy that contributes to the control of pathogen outbreaks, reducing morbidity and mortality. A wide variety of respiratory viruses, including respiratory syncytial virus (hRSV) and metapneumovirus (hMPV), cause serious illness in children under 5 years old and the elderly. To address this public health problem, we have developed recombinant BCG vaccines that have shown to be safe and immunogenic against hRSV or hMPV. Besides the induction of specific adaptive immunity against the viral antigens, these vaccines could generate trained immunity against other respiratory pathogens. Here, we discuss some of the features of trained immunity induced by BCG and put forward the notion that recombinant BCGs expressing hRSV or hMPV antigens have the capacity to simultaneously induce specific adaptive immunity and non-specific trained immunity. These recombinant BCG vaccines could be considered as TIbV capable of inducing simultaneously the development of specific protection against hRSV or hMPV, as well as non-specific trained-immunity-based protection against other pathogenic viruses.

## Introduction

**Figure 1:**
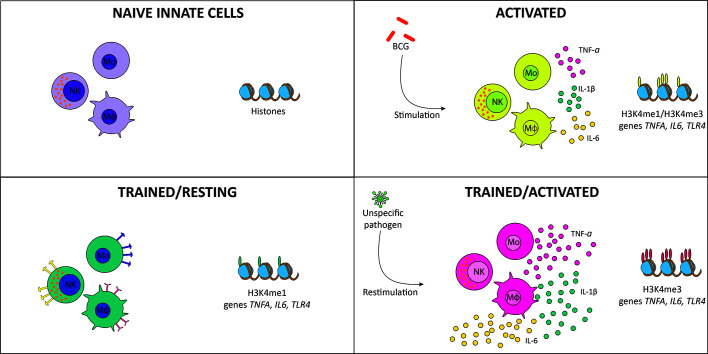
Induction of trained immunity. Naïve monocytes and macrophages (purple) are activated by *Bacillus* Calmette-Guérin (BCG) vaccination, inducing tri-methylation of histones in pro-inflammatory genes and cytokine secretion (yellow). When the infection is resolved, trained cells are maintained in a resting state, with mono-methylation of histones of pro-inflammatory genes and increased expression of membrane receptors (green). When exposed to reinfection, histones are tri-methylated, producing stronger and faster activation and increased cytokine secretion (pink).

Historically, immunological memory development is a characteristic attributed only to the adaptive immune response in an antigen-specific manner. It was recently shown that the innate immune system could develop a type of non-specific immune memory known as “trained immunity” (1). Trained immunity is developed by innate immune cells, such as monocytes, macrophages, and natural killer (NK) cells, after an infection or vaccination (2, 3). Indeed, the development of trained immunity occurs at the hematopoietic stem cells level in the bone marrow, specifically inducing a trained phenotype in myeloid progenitors (4, 5). Epithelial cells can develop a trained phenotype and show an enhanced inflammatory response when exposed to a secondary pathogen, which has been proposed to be associated with epigenetic regulation (6, 7). Trained immunity is induced by β-glucan (8), *Candida albicans* (9), and live vaccines like *Bacillus* Calmette-Guérin (BCG) (10), among others. The exposure to the infectious agent induces innate immune cells to undergo epigenetic modifications in certain pro-inflammatory genes, leading to a “trained” state, which allows the cell to respond in a faster and stronger way against an infection (**Figure 1**) (3, 10).

Trained immunity confers protection against a wide variety of pathogens, including bacteria (11), fungi (3), viruses (12), and protozoan (8). After developing trained immunity in mice, protection is induced against *Escherichia coli, Listeria monocytogenes, Staphylococcus aureus, Citrobacter rodentium*, and *Pseudomonas aeruginosa* (11). In humans, trained monocytes secrete higher levels of interleukin (IL)-1β, Tumor necrosis factor (TNF)-α, and interferon (IFN)-γ when stimulated with *Mycobacterium tuberculosis, S. aureus*, and *C. albicans* as compared to naïve monocytes (3). BCG vaccination before an experimental viral challenge with yellow fever virus, reduces viremia levels due to the development of trained immunity, with a crucial role for IL-1β (12). Moreover, when stimulated with *Leishmania braziliensis*, trained human macrophages secrete higher amounts of pro-inflammatory cytokines compared to naïve macrophages (8). In mice, the induction of trained immunity is sufficient to protect against the infection with *L. braziliensis*, being IL-1β signaling pathways and IL-32 crucial for this protection (8).

The unspecific immunological memory developed by trained innate immune cells can persist at least three months after vaccination (3). Such an effect on the innate immune system persists one year after vaccination, showing IL-1β and TNF-α production levels significantly higher as compared to non-trained cells after *in vitro* stimulation with LPS (13). Also, the fact that trained immunity is developed at hematopoietic stem cell level supports the notion that it might last for extended times *in vivo* (4, 5). Nevertheless, comprehensive long-term studies are necessary to elucidate how long the trained state persists. Importantly, BCG vaccination increases childhood survival during the first five years of life (14, 15), and the heterologous protection induced by this vaccine has been suggested to persist for several years (16).

## Trained Immunity Induced by BCG

Vaccines are designed to induce adaptive immunological memory against specific pathogens (17, 18). However, the recent realization that some vaccines can also induce non-specific immunological memory *via* trained immunity suggests that TIbVs could be considered candidates to protect against pathogens with no specific vaccine. Nevertheless, under what circumstances, non-specific TIbVs could be considered a good strategy for promoting immune protection? A good example are the seasonal outbreaks caused by respiratory viruses that lack commercially available effective vaccines. In this scenario, the ability of some vaccines to promote trained immunity and protect against unrelated pathogens would be relevant to induce an innate immune response that readily works in a regulated manner upon exposure to unrelated pathogens.

The BCG vaccine (an attenuated strain of *M. bovis*) has been widely used for over a century to prevent the disease caused by *M. tuberculosis* (19). Since the beginning of its administration to humans, BCG has reduced the mortality of children due to causes unrelated to *tuberculosis* disease. Therefore, it was proposed that BCG induces the development of a non-specific immune cross-protection based on the generation of trained immunity in humans (16, 20–22). To date, it is not clear how long does the immune protection induced by BCG vaccination lasts. Some studies suggest that it does not protect longer than ten years, while others suggest between 15 to 20 years, and even up 60 years (23–27).

As mentioned above, the first interaction of BCG with the immune system occurs at site of inoculation (28). This interaction begins with resident epidermal macrophages and dendritic cells (DCs) that recognize and phagocyte the bacterium, initiating the immune response (28, 29). DCs phagocyte bacteria and increase their surface expression of activation, maturation, migration, and antigen-presentation molecules (MHC-II, CD40, CD44, CD54, CD80, CD86) (30). Once stimulated, DCs initiate the immune response by secretion of immunomodulatory components, including cytokines and chemokines, such as TNF, IL-1β, IL-6, IL-4, and IL-10 (31). Bacterium-stimulated DCs express on their surface MHC-II molecules loaded with antigenic peptides (32). They migrate from the immunization site through the lymphatic system to the draining lymph nodes where they present antigens to naïve T cells (32). Additionally, circulating neutrophils enter the inoculation site and contribute to the local inflammatory response (28). The interaction of neutrophils with BCG increases the expression of adhesion markers, such as CD11b and CD18, and receptors, including FcγRs II and III and increase the secretion of cytokines and chemokines (e.g., IL-1α, IL-1β, and TGF-β, IL-8, CCL2, and CCL3) (33). Altogether, the interactions between BCG and these immune cells triggers an innate response that will influence the efficacy of this vaccine. As mentioned above, BCG vaccination induces the development of trained immunity (28), which refers to an acquired phenotype developed by innate immune cells after the exposure to live vaccines, including BCG (34–36), measles (35, 37, 38), oral-polio (39, 40), and smallpox (34). This phenotype is also induced by exposure to other pathogenic stimuli, such as *C. albicans* (9), and β-glucan (8). The trained phenotype allows these cells to have a faster and more effective inflammatory response than non-trained ones (1). As shown in **Figure 1**, BCG vaccination induces in monocytes epigenetic modifications in the promoters of several pro-inflammatory genes (3, 10). When the immune system is activated as a consequence of the immunization, histone H3 is mono- and tri-methylated in lysine 4 (H3K4me and H3K4me3, respectively) in the promoters of the pro-inflammatory genes *TNFA, IL6*, and *TLR4*, increasing the accessibility to the transcription machinery and, in consequence, increasing the expression of these genes (1, 3, 10). When the inflammation is resolved and the innate immune cells are resting, H3K4me is conserved in the histones associated with these genes, developing a “trained” state by which the cells can be activated in a reduced time and more efficiently, as compared to naïve or untrained cells (3, 13, 22, 41). When trained cells are re-exposed to an infection, H3K4me3 increases, augmenting the expression of pro-inflammatory genes as well, thus generating a faster and more robust immune response (1, 3, 10). Through the interaction with hematopoietic stem cells, bacteria induce epigenetic modifications that contribute to the training of the monocyte/macrophage linage associated with long-term protection against infection (42). These monocytes and macrophages express higher amounts of PRRs than non-trained cells and exert an enhanced response when exposed to secondary infections (22).

It has been shown that BCG vaccination reduces the risk of developing acute lower respiratory tract infections (ALRI) when exposed to respiratory pathogens (43). Indeed, this study suggests a tendency to reduce the risk of respiratory syncytial virus (hRSV)-associated ALRIs in infants that had received BCG at birth (43). Furthermore, BCG-vaccinated mice show fewer cellular lung infiltrates than non-vaccinated animals after a challenge with either hRSV or metapneumovirus (hMPV) (44, 45). Besides, BCG vaccination promotes antibody secretion (46), probably due to the immunogenic capacity of this attenuated bacterium to induce a strong Th-1 profile (47), promoting the survival and subsequent maturation of B cells population into effector plasma cells, with the induction of antibody secretion upon virus exposure (48). Moreover, BCG vaccination at birth reduces hospitalizations due to respiratory infections in children up to 14 years old (16). Importantly, published data suggest that the unspecific protection induced by BCG vaccination may last for a long time, contrary to previously proposed in other studies (13). Moreover, BCG vaccination increases the plasmatic levels of IFN-γ and prevents acute upper respiratory tract infections (AURTI) in the elderly (49). Indeed, a recently published phase III clinical trial showed that BCG vaccination decreases the prevalence of infections, mostly respiratory, in the elderly (50). Thus, due to the induction of trained immunity, BCG may be an excellent candidate to approach outbreaks caused by respiratory pathogens without commercial vaccines available, such as hRSV, hMPV, parainfluenza, adenovirus, rhinovirus, and coronavirus (51–56). The world is currently living a pandemic caused by a novel severe acute respiratory syndrome coronavirus (SARS-CoV-2) that causes millions of coronavirus disease (COVID-19) cases that can lead to the development of pneumonia and even death (57, 58). Up to date, there is no vaccine nor specific treatment for COVID-19, making it even more difficult to control the spread of this disease (59, 60). As mentioned above, the BCG vaccine induces unspecific protection against respiratory infections, suggesting that trained immunity may represent an interesting strategy to contain the pandemic while specific vaccines are being developed (16, 28, 43, 49). Interestingly, those countries where BCG is included in their national vaccination programs at birth have lower mortality rates than those where it is not included (61–65). Based on these observations, we and others have proposed that BCG vaccination may induce protection against SARS-CoV-2 infection due to the development of trained immunity (65, 66).

BCG exerts a wide variety of beneficial non-specific immunological effects through the induction of trained immunity, ranging from protection against non-mycobacterial infection, decreased incidence of allergic diseases, and increased immunity to certain cancers (67). Because of the ability of BCG to induce an effective immune response, it is used since 1976 for the treatment of bladder cancer (68). Several clinical trials have provided support to the notion that BCG could be a beneficial treatment for this pathology, even though the precise underlying mechanism remains to be elucidated (69–72). Some studies suggest that BCG induces locally a non-specific immune and inflammatory response, contributing to the generation of a localized anti-tumor immunity in patients (73). In a meta-analysis of a randomized clinical trial, BCG was shown to reduce the risk of bladder cancer progression after transurethral resection (74). Also, a topical application of BCG is used as a safe alternative for treating warts in children (75). The immune stimulation in the early years of life induced by BCG vaccination may have a beneficial impact against chronic diseases, such as asthma and allergies (76). Moreover, studies in humans and mice showed that the BCG vaccine offers protection against various viral infections, including herpes and influenza viruses (77). Also, neonatal vaccination protects against sepsis early in life (78). It was recently demonstrated that this latter effect was due to the induction of granulopoiesis by the secretion of Granulocyte colony-stimulating factor (G-CSF), resulting in neutrophil expansion (78). These BCG-induced neutrophils were shown to be necessary and sufficient to induce such protection against sepsis (78). Further, it was proposed that a “trained” innate immune system can direct the adaptive immune response towards a more effective response against different pathogens (77, 79). The enhanced activation of the innate immune system and the secretion of high levels of IL-1β by these cells, when exposed to an infectious agent, may activate more effectively the adaptive immune response (80). Besides, the increased secretion of pro-inflammatory cytokines can accelerate the maturation of DCs, which represent the direct cross-talk between the innate and adaptive immune responses (81–83). Indeed, DCs cooperate with neutrophils after BCG-infection to stimulate T cell responses against these bacteria (84). On the other hand, trained immunity generated by BCG induces heterologous Th1 and Th17 responses, characterized by the secretion of IFN-γ and IL-17, and IL-22, respectively (80). Altogether these characteristics of a trained innate immune response can induce a more effective adaptive immune response against the pathogen. In agreement with this statement, vaccination with BCG before the administration of a trivalent influenza vaccine improves the specific antibody response, inducing a faster seroconversion compared to the administration of the influenza vaccine by itself (85). This finding further supports the notion that trained innate immune cells may activate a more effective adaptive immune response.

Although the non-specific immune effects induced by the BCG vaccine are broadly reported, the molecular mechanisms involved in this phenomenon are only partially understood. Unveiling these mechanisms would be important to design better therapeutic options and vaccination strategies using this attenuated bacterium.

## Trained Immunity Induced by Recombinant Vaccines

Worldwide, hRSV is considered the most important etiologic agent of acute lower respiratory tract infections (ALRIs) in children under 5 years and adults over 65 years (86), infecting 100% of children at age two (87). In 2015, hRSV caused 33.1 million episodes of ALRIs (bronchiolitis and pneumonia) worldwide, producing 3.2 million hospitalizations and about 120,000 deaths of children under 5 years old (88).

Vaccines consisting of recombinant strains of BCG expressing either the nucleoprotein (N) or the M2 protein of hRSV (rBCG-N-hRSV and rBCG-M2-hRSV, respectively) were evaluated to induce hRSV specific immunity and prevent disease (44, 45, 89–92). These formulations were shown to protect against hRSV in a murine model of infection, reducing the development of clinical symptoms associated with the viral challenge in vaccinated mice (44). The rBCG-N-hRSV and rBCG-M2-hRSV vaccines induce the development of cellular and humoral responses, generating specific T_H1_/T_H17_ memory cells and antibodies in mice (44, 45, 89, 91). Viral-specific antibodies have neutralizing activity (45), which correlates with diminished viral titers in the lungs of immunized animals (89). rBCG-N-hRSV was developed under cGMP *(Good Manufacturing Practices)* conditions, showing the same protection against hRSV infection mentioned above in animal models (89). Indeed, this vaccine is the only hRSV-vaccine being developed to be administered to newborns, who represent the major risk group for this virus (93). In a phase 1 clinical trial, rBCG-N-hRSV was shown to induce both cellular and humoral immunity against hRSV in humans (90).

HMPV also causes ALRIs and death in children under 5 years old (94). This virus was first described in 2001 by Van Den Hoogen et al. (95), and its incidence has increased every year since then (96, 97). In healthy adults, hMPV infection appears with mild influenza-like symptoms, while in children under 5 years old, elderly, and immunocompromised patients it causes bronchiolitis, pneumonia, and even death (96).

Based on the previous experience with hRSV, recombinant BCG vaccines expressing either the phosphoprotein (P) or the M2.1 protein of hMPV were generated (98). Both, rBCG-P-hMPV and rBCG-M2.1-hMPV, were shown to efficiently protect from hMPV in a murine model of infection, with less cellular infiltrates, lung inflammation, and viral replication in vaccinated mice (45, 98). Mouse vaccination was also effective in the induction of humoral responses against hMPV, with virus-neutralizing antibody production and isotype switching (45).

These recombinant BCG vaccines were based on BCG-Danish 1331, a vaccine that is known to induce trained immunity, suggesting that this type of immunity might be a component of the protection achieved with these vaccines against these two respiratory viruses. Consistently with this notion, vaccination of RAG-deficient mice, which lack T and B cells, with rBCG-N-hRSV induces the secretion of significantly higher IFN-γ levels in bronchioalveolar lavages (BAL) after hRSV challenge as compared to unimmunized mice (91). Surprisingly, this induction reached similar levels of IFN-γ as BCG-WT, suggesting that both could induce a trained immunity phenotype in these immunodeficient mice (91). Furthermore, immunization with BCG-WT reduced cellular lung infiltration and inflammation in murine models for both hRSV and hMPV infections (44). BCG-WT induces specific antibody isotype switching and the production of neutralizing antibodies after hRSV and hMPV infections in mice (45). These data suggest that non-specific cross-protection induced by BCG may be effective in protecting against these viruses.

During viral infections, the immune system activates the production and secretion of interferons, which mediate the antiviral response, impairing the viral replication by the activation of macrophages and DC (99). As mentioned above, rBCG-N-hRSV and rBCG-P-hMPV vaccination generated an early IFN production soon after the viral challenge in mice, suggesting that these vaccines may induce a non-specific cross-protection against other viral infections (91, 98). The induction of trained immunity-related cytokines, such as IL-6, TNF, and IL-1β by these vaccines still has to be elucidated (3). Besides hRSV and hMPV, there are other important respiratory viruses, as parainfluenza, adenovirus, rhinovirus, and the newly identified SARS-CoV-2, among others (51–56, 100). The unspecific protection mediated by the development of trained immunity may be a good strategy to protect against a broad spectrum of viruses, being recombinant BCGs excellent candidates, since BCG is a safe vaccine used to immunize infants, which constitute a high-risk population (101). Trained immunity protects against yellow fever virus infection in humans (12). In an experimental infection challenge of healthy volunteers, BCG vaccination reduced the viremia after infection compared to non-vaccinated volunteers (12).

**Figure 2:**
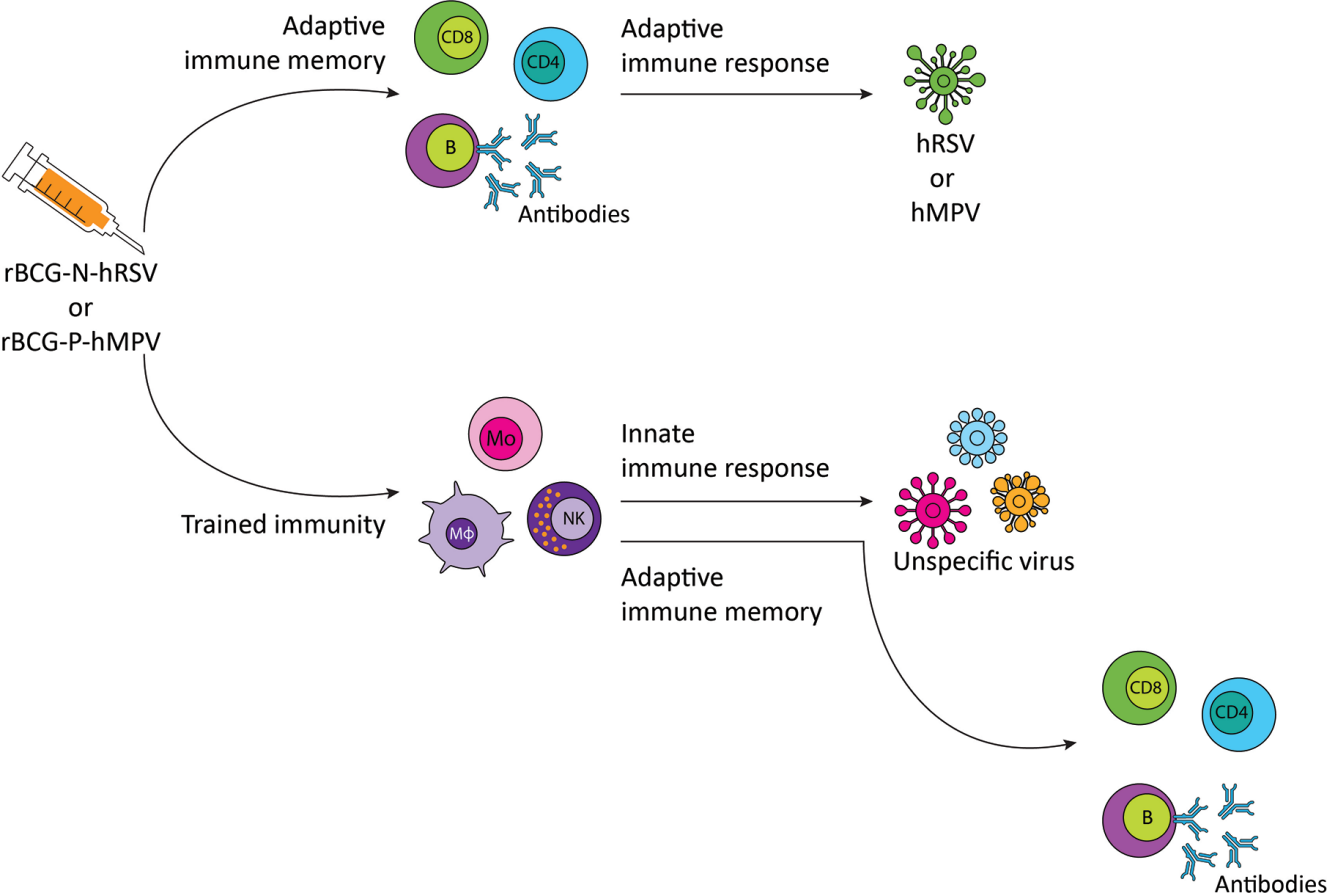
Proposed immune response to respiratory syncytial virus (hRSV) or metapneumovirus (hMPV) and non-specific viruses after rBCG-N-hRSV or rBCG-P-hMPV vaccination, respectively. Immunization with rBCG-N-hRSV or rBCG-P-hMPV induces a specific adaptive immune response against hRSV or hMPV, respectively, that will resolve the infection. Trained immunity induction generates a more robust response against other viruses and orchestrates adaptive immune memory against the virus. CD8, CD8^+^ T cell; CD4, CD4^+^ T cell; B, B cell; Mo, monocyte; Mφ, macrophage; NK, natural killer.

Based on the studies mentioned above, vaccination of the risk population with either rBCG-N-hRSV or rBCG-P-hMPV, besides inducing specific protection against the virus for which they are developed, may induce non-specific cross-protection mediated by a trained innate immune system against other viruses to which there is no specific vaccine available (**Figure 2**).

In the context of the pandemic that the world is currently facing, it has been proposed that SARS-CoV-2 exposure in the presence of a trained innate immune system may induce a more robust and effective immune response and, in consequence, a milder manifestation of the disease (65, 102–106). Indeed, those countries where BCG is included in their national vaccination programs at birth showed lower mortality rates due to SARS-CoV-2 infection as compared that do not include it (61, 62, 65, 107, 108), probably by the development of trained immunity. Based on this hypothesis, several clinical trials are being performed in different countries, including Netherlands, South Africa, Australia, United States, Colombia, Egypt, Brazil, Denmark, and France (Clinicaltrials.gov) (104). Interestingly, a recombinant BCG vaccine, VPM1002, is also being tested in a clinical trial to determine the cross-protection against COVID-19 (Clinicaltrials.gov ID: NCT04387409) (104). This recombinant BCG has a *Listeria monocytogenes* gene encoded for listeriolysin instead of the urease C gene (109). This modification increases apoptosis and autophagy, promotes phagolysosome fusion, and improves vaccine efficacy (109).

Even though the induction of trained immunity has not yet been demonstrated for recombinant BCG vaccines, some studies support this notion and suggest an advantageous scenario for the prevention of several infections (28). Positive results deriving from the VPM1002 clinical trial, will suggest a cross-protection due to the induction of trained immunity.

Besides the intramuscular administration of live-attenuated BCG to induce protection against tuberculosis, this bacterium can be inactivated and used as an adjuvant based on its strong immunogenicity (110–112). Also, inactivated BCG induces the development of trained immunity in innate immune cells *in vitro* (10). Based on these findings, a vaccine formulation containing inactivated BCG as an adjuvant could promote the development of a strong specific immune response and induce the development of trained immunity. On the other hand, to prevent and treat respiratory infections, a sublingual vaccine was designed consisting of heat-inactivated bacterial components, called MV130 (113). This vaccine was shown to trigger TLR and NLR signaling pathways on DCs, inducing the production and secretion of trained immunity-related cytokines, such as TNF-α, IL-6, and IL-1β (113). These bacterial preparations could also be considered as good adjuvants for the development of novel vaccines with the capacity to induce trained immunity.

## Concluding Remarks

Vaccines have been used since 1798 to control infections that are potentially harmful to humans (17). A very important feature of vaccines is the specificity of the immunological memory that they induce against a unique pathogen (114). However, what happens when there are no efficient vaccines to protect us against a particular infectious agent? Trained immunity is a non-specific immunological memory mediated by the innate immune system (1). This type of immunological memory protects against a wide variety of pathogens, suggesting that it could be considered an alternative for developing unspecific vaccines (3, 8, 11, 12).

As mentioned above, BCG protects against some respiratory affections, including asthma and upper and lower respiratory tract infections non-related to *M. tuberculosis* (12, 43, 48, 49, 77). These characteristics of BCG are attributed to the induction of trained immunity (1, 28). Similar to BCG, recombinant BCG formulations are expected to induce trained immunity (28). We have developed effective, immunogenic rBCG vaccines against hRSV and hMPV, two common respiratory viruses that can cause serious illness, even death (44, 45, 89, 91, 98). We are exposed to a wide variety of viruses, many of them with no specific vaccine or antiviral drugs available to treat them (100). As proposed in **Figure 2**, an immunogenic vaccine with the ability to induce specific immunity against two of the most relevant respiratory viruses (hRSV or hMPV) and trained immunity to other seasonal respiratory viruses could be an approach to be evaluated for controlling outbreaks and reduce the morbidity and mortality associated to other respiratory viral infections. Up to date, the induction of trained immunity by recombinant BCGs has not been demonstrated. Even though some studies suggest that these vaccines have the same capacity as the parental BCG strain to induce this non-specific innate memory, a formal demonstration would require additional research.

## Author Contributions

CC, MR, and RB-R wrote the manuscript. AK and SB reviewed the manuscript and approved the version to be published. All authors contributed to the article and approved the submitted version.

## Funding

This research was funded by CONICYT PAI project I781902009 Chile, as well as the Millennium Institute on Immunology and Immunotherapy grant number P09/016-F and ICN09_016. The Regional Government of Antofagasta also supported this work through the Innovation Fund for Competitiveness FIC- R 2017 (BIP Code: 30488811-0). FONDECYT N°1170964 and 1190830. AK is a Helen C. Levitt visiting professor at the Department of Microbiology and Immunology of the University of Iowa.

## Conflict of Interest

The authors declare that the research was conducted in the absence of any commercial or financial relationships that could be construed as a potential conflict of interest.

## References

[1] Netea MG Quintin J Van Der Meer JWM Trained immunity: A memory for innate host defense. Cell Host Microbe (2011) 9(5):355–61. 10.1016/j.chom.2011.04.006 21575907

[2] Sun JC Lopez-Verges S Kim CC DeRisi JL Lanier LL NK cells and immune “memory”. J Immunol (2011) 186(4):1891–7. 10.4049/jimmunol.1003035 PMC4410097 21289313

[3] Kleinnijenhuis J Quintin J Preijers F Joosten LAB Ifrim DC Saeed S Bacille Calmette-Guerin induces NOD2-dependent nonspecific protection from reinfection via epigenetic reprogramming of monocytes. Proc Natl Acad Sci (2012) 109(43):17537–42. 10.1073/pnas.1202870109 PMC3491454 22988082

[4] Mitroulis I Ruppova K Wang B Chen LS Grzybek M Grinenko T Modulation of Myelopoiesis Progenitors Is an Integral Component of Trained Immunity. Cell (2018) 172(1–2):147–61.e12. 10.1016/j.cell.2017.11.034 29328910 PMC5766828

[5] Kaufmann E Sanz J Dunn JL Khan N Mendonça LE Pacis A BCG Educates Hematopoietic Stem Cells to Generate Protective Innate Immunity against Tuberculosis. Cell (2018) 172(1–2):176–90.e19. 10.1016/j.cell.2017.12.031 29328912

[6] Novakovic B Stunnenberg HG I Remember You: Epigenetic Priming in Epithelial Stem Cells. Immunity (2017) 47(6):1019–21. 10.1016/j.immuni.2017.12.005 29262345

[7] Naik S Larsen SB Gomez NC Alaverdyan K Sendoel A Yuan S Inflammatory memory sensitizes skin epithelial stem cells to tissue damage. Nature (2017) 550(7677):475–80. 10.1038/nature24271 PMC5808576 29045388

[8] dos Santos JC Barroso de Figueiredo AM Teodoro Silva MV Cirovic B de Bree LCJ Damen MSMA β-Glucan-Induced Trained Immunity Protects against Leishmania braziliensis Infection: a Crucial Role for IL-32. Cell Rep (2019) 28(10):2659–72.e6. 10.1016/j.celrep.2019.08.004 31484076

[9] Quintin J Saeed S Martens JHA Giamarellos-Bourboulis EJ Ifrim DC Logie C Candida albicans infection affords protection against reinfection via functional reprogramming of monocytes. Cell Host Microbe (2012) 12(2):223–32. 10.1016/j.chom.2012.06.006 PMC3864037 22901542

[10] Arts RJW Blok BA Aaby P Joosten LAB de Jong D van der Meer JWM Long-term in vitro and in vivo effects of γ-irradiated BCG on innate and adaptive immunity. J Leukoc Biol (2015) 98(6):995–1001. 10.1189/jlb.4MA0215-059R 26082519

[11] Ciarlo E Heinonen T Théroude C Asgari F Le Roy D Netea MG Trained Immunity Confers Broad-Spectrum Protection Against Bacterial Infections. J Infect Dis (2020) 222(11):1869–81. 10.1093/infdis/jiz692 PMC7653089 31889191

[12] Arts RJW Moorlag SJCFM Novakovic B Li Y Wang SY Oosting M BCG Vaccination Protects against Experimental Viral Infection in Humans through the Induction of Cytokines Associated with Trained Immunity. Cell Host Microbe (2018) 23(1):89–100.e5. 10.1016/j.chom.2017.12.010 29324233

[13] Kleinnijenhuis J Quintin J Preijers F Benn CS Joosten LAB Jacobs C Long-lasting effects of BCG vaccination on both heterologous Th1/Th17 responses and innate trained immunity. Innate Immun (2015) 6: (2):152–8. 10.1159/000355628 PMC3944069 24192057

[14] Netea MG Domínguez-Andrés J Barreiro LB Chavakis T Divangahi M Fuchs E Defining trained immunity and its role in health and disease. Nat Rev Immunol (2020) 20(6):375–88. 10.1038/s41577-020-0285-6 PMC7186935 32132681

[15] Nankabirwa V Tumwine JK Mugaba PM Tylleskär T Sommerfelt H Van De Perre P Child survival and BCG vaccination: A community based prospective cohort study in Uganda. BMC Public Health (2015) 15(1):1–10. 10.1186/s12889-015-1497-8 25886062 PMC4342809

[16] De Castro MJ Pardo-Seco J Martinón-Torres F Nonspecific (heterologous) protection of neonatal BCG vaccination against hospitalization due to respiratory infection and sepsis. Clin Infect Dis (2015) 60(11):1611–9. 10.1093/cid/civ144 25725054

[17] Rey-Jurado E Tapia F Muñoz-Durango N Lay MK Carreño LJ Riedel CA Assessing the Importance of Domestic Vaccine Manufacturing Centers: An Overview of Immunization Programs, Vaccine Manufacture, and Distribution. Front Immunol (2018) 9:26. 10.3389/fimmu.2018.00026 29403503 PMC5778105

[18] Rolling KE Hayney MS The vaccine development process. J Am Pharm Assoc (2016) 56(6):687–9. 10.1016/j.japh.2016.09.009 27836129

[19] WHO Global World Health Organization. (2018). Available at: https://www.who.int/tb/publications/global_report/en/.

[20] De Bree LCJ Koeken VACM Joosten LAB Aaby P Benn CS van Crevel R Non-specific effects of vaccines: Current evidence and potential implications. Semin Immunol (2018) 39(April):35–43. 10.1016/j.smim.2018.06.002 30007489

[21] Aaby P Benn CS Saving lives by training innate immunity with bacille Calmette-Guérin vaccine. Proc Natl Acad Sci USA (2012) 109: (43):17317–8. 10.1073/pnas.1215761109 PMC3491466 23071307

[22] Kleinnijenhuis J van Crevel R Netea MG Trained immunity: consequences for the heterologous effects of BCG vaccination. Trans R Soc Trop Med Hyg (2015) 109(1):29–35. 10.1093/trstmh/tru168 25573107

[23] Barreto ML Cunha SS Pereira SM Genser B Hijjar MA Ichihara MY Neonatal BCG protection against tuberculosis lasts for 20 years in Brazil. Int J Tuberc Lung Dis (2005) 9(10):1171–3. 16229231

[24] Rodrigues LC Mangtani P Abubakar I How does the level of BCG vaccine protection against tuberculosis fall over time? BMJ (2011) 343(7826):1–3. 10.1136/bmj.d5974 21964347

[25] Russell DG Barry CE Flynn JL Tuberculosis: What We Don ‘ t Know. Sci (80- ) (2010) 328(5980):852–6. 10.1126/science.1184784 PMC2872107 20466922

[26] Aronson NE Santosham M Comstock GW Howard RS Moulton LH Rhoades ER Long-term efficacy of BCG vaccine. J Am Med Assoc (2004) 291(17):2086. 10.1001/jama.291.17.2086 15126436

[27] Hart PD Sutherland I BCG and vole bacillus vaccines in the prevention of tuberculosis in adolescence and early adult life. Br Med J (1977) 2(6082):293–5. 10.1136/bmj.2.6082.293 PMC1630784 326347

[28] Covián C Fernández-Fierro A Retamal-Díaz A Díaz FE Vasquez AE Lay MK BCG-Induced Cross-Protection and Development of Trained Immunity: Implication for Vaccine Design. Front Immunol (2019) 10(November):1–14. 10.3389/fimmu.2019.02806 31849980 PMC6896902

[29] Moliva JI Turner J Torrelles JB Immune Responses to Bacillus Calmette–Guérin Vaccination: Why Do They Fail to Protect against Mycobacterium tuberculosis? Front Immunol (2017) 8:407. 10.3389/fimmu.2017.00407/full 28424703 PMC5380737

[30] Kim KD Lee HG Kim JK Park SN Choe IS Choe YK Enhanced antigen-presenting activity and tumour necrosis factor-α- independent activation of dendritic cells following treatment with Mycobacterium bovis bacillus Calmette-Guerin. Immunology (1999) 97(4):626–33. 10.1046/j.1365-2567.1999.00818.x PMC2326884 10457216

[31] Sendide K Deghmane A-E Pechkovsky D Av-Gay Y Talal A Hmama Z Mycobacterium bovis BCG Attenuates Surface Expression of Mature Class II Molecules through IL-10-Dependent Inhibition of Cathepsin S. J Immunol (2005) 175(8):5324–32. 10.4049/jimmunol.175.8.5324 16210638

[32] Demangel C Bean AGD Martin E Feng CG Kamath AT Britton WJ Protection against aerosol Mycobacterium tuberculosis infection using Mycobacterium bacillus Calmette Guerin-infected dendritic cells. Int J Lepr Other Mycobact Dis (1999) 67(4 SUPPL.):502. 10.1002/(SICI)1521-4141(199906)29:06<1972::AID-IMMU1972>3.0.CO;2-1 10382760

[33] Suttmann H Lehan N Böhle A Brandau S Stimulation of Neutrophil Granulocytes with Mycobacterium bovis Bacillus Calmette-Guérin Induces Changes in Phenotype and Gene Expression and Inhibits Spontaneous Apoptosis. Infect Immun (2003) 71(8):4647–56. 10.1128/IAI.71.8.4647-4656.2003 PMC165987 12874345

[34] Rieckmann A Villumsen M Sørup S Haugaard LK Ravn H Roth A Vaccinations against smallpox and tuberculosis are associated with better long-term survival: A Danish case-cohort study 1971-2010. Int J Epidemiol (2017) 46(2):695–705. 10.1093/ije/dyw120 27380797 PMC5837789

[35] Aaby P Gustafson P Roth A Rodrigues A Fernandes M Sodemann M Vaccinia scars associated with better survival for adults. An observational study from Guinea-Bissau. Vaccine (2006) 24(29–30):5718–25. 10.1016/j.vaccine.2006.04.045 16720061

[36] Aaby P Roth A Ravn H Napirna BM Rodrigues A Lisse IM Randomized Trial of BCG Vaccination at Birth to Low-Birth-Weight Children: Beneficial Nonspecific Effects in the Neonatal Period? J Infect Dis (2011) 204:245–52. 10.1093/infdis/jir240 21673035

[37] Aaby P Samb B Simondon F Seck AMC Knudsen K Whittle H Non-specific beneficial effect of measles immunisation: Analysis of mortality studies from developing countries. BMJ (1995) 311(7003):481. 10.1136/bmj.311.7003.481 7647643 PMC2550544

[38] Aaby P Martins CL Garly ML Balé C Andersen A Rodrigues A Non-specific effects of standard measles vaccine at 4.5 and 9 months of age on childhood mortality: Randomised controlled trial. BMJ (2010) 341(7785):1262. 10.1136/bmj.c6495 PMC2994348 21118875

[39] Andersen A Fisker AB Rodrigues A Martins C Ravn H Lund N National Immunization Campaigns with Oral Polio Vaccine Reduce All-Cause Mortality: A Natural Experiment within Seven Randomized Trials. Front Public Heal (2018) 6(February):1–10. 10.3389/fpubh.2018.00013 PMC5801299 29456992

[40] Lund N Andersen A Hansen ASK Jepsen FS Barbosa A Biering-Sørensen S The Effect of Oral Polio Vaccine at Birth on Infant Mortality: A Randomized Trial. Clin Infect Dis (2015) 61(10):1504–11. 10.1093/cid/civ617 PMC4614411 26219694

[41] Kleinnijenhuis J Quintin J Preijers F Joosten LAB Jacobs C Xavier RJ BCG-induced trained immunity in NK cells: Role for non-specific protection to infection. Clin Immunol (2014) 155(2):213–9. 10.1016/j.clim.2014.10.005 PMC5084088 25451159

[42] Kaufmann E Sanz J Dunn JL Khan N Mendonça LE Pacis A BCG Educates Hematopoietic Stem Cells to Generate Protective Innate Immunity against Tuberculosis. Cell (2018) 172(1–2):176–90.e19. 10.1016/j.cell.2017.12.031 29328912

[43] Stensballe LG Nante E Jensen IP Kofoed P-E Poulsen A Jensen H Acute lower respiratory tract infections and respiratory syncytial virus in infants in Guinea-Bissau: a beneficial effect of BCG vaccination for girls. Vaccine (2005) 23(10):1251–7. 10.1016/j.vaccine.2004.09.006 15652667

[44] Bueno SM Gonzalez PA Cautivo KM Mora JE Leiva ED Tobar HE Protective T cell immunity against respiratory syncytial virus is efficiently induced by recombinant BCG. Proc Natl Acad Sci (2008) 105(52):20822–7. 10.1073/pnas.0806244105 PMC2634951 19075247

[45] Soto JA Gálvez NMS Rivera CA Palavecino CE Céspedes PF Rey-Jurado E Recombinant BCG Vaccines Reduce Pneumovirus-Caused Airway Pathology by Inducing Protective Humoral Immunity. Front Immunol (2018) 9(December):2875. 10.3389/fimmu.2018.02875 30581437 PMC6293239

[46] Patel AA Zhang Y Fullerton JN Boelen L Rongvaux A Maini AA The fate and lifespan of human monocyte subsets in steady state and systemic inflammation. J Exp Med (2017) 214(7):1913–23. 10.1084/jem.20170355 PMC5502436 28606987

[47] Finkelman FD Holmes J Lymphokine control of in vivo immunoglobulin isotype selection. Annu Rev Immunol (1990) 8:303–33. 10.1146/annurev.iy.08.040190.001511 1693082

[48] Drowart A Selleslaghs J Yernault J Valcke C De Bruyn J Huygen K The humoral immune response after BCG vaccination: an immunoblotting study using two purified antigens. Tuber Lung Dis (1992) 73:137–40. 10.1016/0962-8479(92)90146-B 1421345

[49] Wardhana, Datau EA Sultana A Mandang VV Jim E The efficacy of Bacillus Calmette-Guerin vaccinations for the prevention of acute upper respiratory tract infection in the elderly. Acta Med Indones (2011) 43(3):185–90. 21979284

[50] Giamarellos-Bourboulis EJ Tsilika M Moorlag S Antonakos N Kotsaki A Domínguez-Andrés J Activate: Randomized Clinical Trial of BCG Vaccination against Infection in the Elderly. Cell (2020) 183(2):315–23.e9. 10.1016/j.cell.2020.08.051 32941801 PMC7462457

[51] Su IC Lee KL Liu HY Chuang HC Chen LY Lee YJ Severe community-acquired pneumonia due to Pseudomonas aeruginosa coinfection in an influenza A(H1N1)pdm09 patient. J Microbiol Immunol Infect (2019) 52(2):365–6. 10.1016/j.jmii.2018.05.007 29958866

[52] Lee KH Yoo SG Cho Y Kwon DE La Y Han SH Characteristics of community-acquired respiratory viruses infections except seasonal influenza in transplant recipients and non-transplant critically ill patients J Microbiol Immunol Infect. J Microbiol Immunol Infect [Preprint] (2019). 10.1016/j.jmii.2019.05.007. (xxxx). PMC7102620 31262511

[53] Chou CC Shen CF Chen SJ Chen HM Wang YC Chang WS Recommendations and guidelines for the treatment of pneumonia in Taiwan. J Microbiol Immunol Infect (2019) 52(1):172–99. 10.1016/j.jmii.2018.11.004 30612923

[54] Lee JY Yang PC Chang C Lin IT Ko WC Cia CT Community-acquired adenoviral and pneumococcal pneumonia complicated by pulmonary aspergillosis in an immunocompetent adult. J Microbiol Immunol Infect (2019) 52(5):838–9. 10.1016/j.jmii.2019.05.014 31337539

[55] Lai C Shih T Ko W Tang H Hsueh P Severe acute respiratory syndrome coronavirus 2 ( SARS-CoV-2 ) and coronavirus disease-2019 ( COVID-19 ): The epidemic and the challenges. Int J Antimicrob Agents (2020) 55(3):105924. 10.1016/j.ijantimicag.2020.105924 32081636 PMC7127800

[56] Feuillet F Lina B Rosa-Calatrava M Boivin G Ten years of human metapneumovirus research. J Clin Virol (2012) 53(2):97–105. 10.1016/j.jcv.2011.10.002 22074934

[57] Huang Y Zhou H Yang R Xu Y Feng X Gong P Clinical characteristics of 36 non-survivors with COVID-19 in Wuhan, China. medRxiv [Preprint] (2020). 10.1101/2020.02.27.20029009v1. 2020.02.27.20029009.

[58] Moghadas SM Temporal estimates of case-fatality rate for COVID-19 outbreaks in Canada and the United States. CMAJ [Preprint] (2020). 10.1503/cmaj.200711 PMC7828851 32444481

[59] Callaway E The race for coronavirus vaccines. Nature (2020) 580:576–7. 10.1038/d41586-020-01221-y 32346146

[60] Thanh Le T Andreadakis Z Kumar A Gómez Román R Tollefsen S Saville M The COVID-19 vaccine development landscape. Nat Rev Drug Discovery (2020) 19(May):305–6. 10.1038/d41573-020-00073-5 32273591

[61] Berg MK Yu Q Salvador CE Melani I Kitayama S Mandated Bacillus Calmette-Guérin (BCG) vaccination predicts flattened curves for the spread of COVID-19. Sci Adv (2020) 6(32):eabc1463. 10.1126/sciadv.abc1463 32923613 PMC7457335

[62] Jirjees FJ Dallal Bashi YH Al-Obaidi HJ COVID-19 death and BCG vaccination programs worldwide. Tuberc Respir Dis (Seoul) [Preprint] (2020). 10.4046/trd.2020.0063 PMC7801810 32883062

[63] Wickramasinghe D Wickramasinghe N Kamburugamuwa SA Arambepola C Samarasekera DN Correlation between immunity from BCG and the morbidity and mortality of COVID-19. Trop Dis Travel Med Vaccines (2020) 6(1):1–8. 10.1186/s40794-020-00117-z 32868985 PMC7453689

[64] Ebina-Shibuya R Horita N Namkoong H Kaneko T Current national policies for infant universal bacille calmetteguérin vaccination were associated with lower mortality from coronavirus disease 2019. Clin Exp Vaccine Res (2020) 9(2):179–82. 10.7774/cevr.2020.9.2.179 PMC7445316 32864376

[65] Covián C Retamal-Diaz A Bueno SM Kalergis AM Could BCG vaccination induce protective trained immunity for SARS-CoV-2? Front Immunol (2020) 11(May):970. 10.3389/fimmu.2020.00970 32574258 PMC7227382

[66] Miller A Reandelar MJ Fasciglione K Roumenova V Li Y Otazu GH Correlation between universal BCG vaccination policy and reduced morbidity and mortality for COVID-19: an epidemiological study. medRxiv [Preprint] (2020) 2020.03.24.20042937. 10.3113/JSOA.2020.0036

[67] Netea MG Van Crevel R BCG-induced protection: Effects on innate immune memory. Semin Immunol (2014) 26(6):512–7. 10.1016/j.smim.2014.09.006 25444548

[68] Morales A Eidinger D Bruce AW Intracavitary Bacillus Calmette-Guerin in the treatment of superficial bladder tumors. J Urol (1976) 116(2):180–3. 10.1016/S0022-5347(17)58737-6 820877

[69] Porena M Del Zingaro M Lazzeri M Mearini L Giannantoni A Bini V Bacillus calmette-guérin versus gemcitabine for intravesical therapy in high-risk superficial bladder cancer: A randomised prospective study. Urol Int (2010) 84(1):23–7. 10.1159/000273461 20173364

[70] Rentsch CA Birkhäuser FD Biot C Gsponer JR Bisiaux A Wetterauer C Bacillus calmette-guérin strain differences have an impact on clinical outcome in bladder cancer immunotherapy. Eur Urol (2014) 66(4):677–88. 10.1016/j.eururo.2014.02.061 24674149

[71] Duchek M Johansson R Jahnson S Mestad O Hellström P Hellsten S Bacillus Calmette-Guérin Is Superior to a Combination of Epirubicin and Interferon-α2b in the Intravesical Treatment of Patients with Stage T1 Urinary Bladder Cancer. A Prospective, Randomized, Nordic Study. Eur Urol (2010) 57(1):25–31. 10.1016/j.eururo.2009.09.038 19819617

[72] Yokomizo A Kanimoto Y Okamura T Ozono S Koga H Iwamura M Randomized Controlled Study of the Efficacy, Safety and Quality of Life with Low Dose bacillus Calmette-Guérin Instillation Therapy for Nonmuscle Invasive Bladder Cancer. J Urol (2016) 195(1):41–6. 10.1016/j.juro.2015.08.075 26307162

[73] Wu Z Liu J Dai R Wu S Current status and future perspectives of immunotherapy in bladder cancer treatment. Sci China Life Sci (2020). 10.1007/s11427-020-1768-y 32926318

[74] Sylvester RJ van der Meijden APM Lamm DL Intravesical Bacillus Calmette-Guerin Reduces the Risk of Progression in Patients With Superficial Bladder Cancer: A Meta-Analysis of the Published Results of Randomized Clinical Trials. J Urol (2002) 168(5):1964–70. 10.1016/S0022-5347(05)64273-5 12394686

[75] Salem A Nofal A Hosny D Treatment of common and plane warts in children with topical viable bacillus calmette-guerin. Pediatr Dermatol (2013) 30(1):60–3. 10.1111/j.1525-1470.2012.01848.x 22958215

[76] Rousseau MC Parent ME St-Pierre Y Potential health effects from non-specific stimulation of the immune function in early age: The example of BCG vaccination. Pediatr Allergy Immunol (2008) 19(5):438–48. 10.1111/j.1399-3038.2007.00669.x 18167158

[77] Moorlag SJCFM Arts RJW van Crevel R Netea MG Non-specific effects of BCG vaccine on viral infections. Clin Microbiol Infect (2019) 25(12):1473–8. 10.1016/j.cmi.2019.04.020 31055165

[78] Brook B Harbeson DJ Shannon CP Cai B He D Ben-othman R BCG vaccination–induced emergency granulopoiesis provides rapid protection from neonatal sepsis. Sci Transl Med (2020) 12(4517):1–15. 10.1126/scitranslmed.aax4517 PMC8008103 32376769

[79] Sánchez-Ramón S Conejero L Netea MG Sancho D Palomares Ó Subiza JL Trained Immunity-Based Vaccines: A New Paradigm for the Development of Broad-Spectrum Anti-infectious Formulations. Front Immunol (2018) 9:2936. 10.3389/fimmu.2018.02936 30619296 PMC6304371

[80] Kleinnijenhuis J Quintin J Preijers F Benn CS Joosten LAB Jacobs C Long-lasting effects of bcg vaccination on both heterologous th1/th17 responses and innate trained immunity. J Innate Immun (2014) 6(2):152–8. 10.1159/000355628 PMC3944069 24192057

[81] Zanoni I Tan Y Di Gioia M Broggi A Ruan J Shi J An endogenous caspase-11 ligand elicits interleukin-1 release from living dendritic cells. Sci (80- ) (2016) 352(6290):1232–6. 10.1126/science.aaf3036 PMC5111085 27103670

[82] Su H Peng B Zhang Z Liu Z Zhang Z The Mycobacterium tuberculosis glycoprotein Rv1016c protein inhibits dendritic cell maturation, and impairs Th1/Th17 responses during mycobacteria infection. Mol Immunol (2019) 109:58–70. 10.1016/j.molimm.2019.02.021 30856410

[83] Bollampalli VP Yamashiro LH Feng X Bierschenk D Gao Y Blom H BCG Skin Infection Triggers IL-1R-MyD88- Dependent Migration of EpCAM low CD11b high Skin Dendritic cells to Draining Lymph Node During CD4 + T-Cell Priming. PLoS Pathog (2015) 11(10):1–22. 10.1371/journal.ppat.1005206 PMC4594926 26440518

[84] Morel C Badell E Abadie V Robledo M Setterblad N Gluckman JC Mycobacterium bovis BCG-infected neutrophils and dendritic cells cooperate to induce specific T cell responses in humans and mice. Eur J Immunol (2008) Dc):437–47. 10.1002/eji.200737905 18203135

[85] Leentjens J Kox M Stokman R Gerretsen J Diavatopoulos DA van Crevel R BCG-vaccination enhances immunogenicity of subsequent influenza vaccination in healthy volunteers: a randomized placebo-controlled pilot study. J Infect Dis (2015) 212(12):1930–8. 10.1093/infdis/jiv332 26071565

[86] Bont L Checchia PA Fauroux B Figueras-Aloy J Manzoni P Paes B Defining the Epidemiology and Burden of Severe Respiratory Syncytial Virus Infection Among Infants and Children in Western Countries. Infect Dis Ther (2016) 5(3):271–98. 10.1007/s40121-016-0123-0 PMC5019979 27480325

[87] Nair H Nokes DJ Gessner BD Dherani M Madhi SA Singleton RJ Global burden of acute lower respiratory infections due to respiratory syncytial virus in young children: a systematic review and meta-analysis. Lancet (2010) 375(9725):1545–55. 10.1016/S0140-6736(10)60206-1 PMC2864404 20399493

[88] Shi T McAllister DA O’Brien KL Simoes EAF Madhi SA Gessner BD Global, regional, and national disease burden estimates of acute lower respiratory infections due to respiratory syncytial virus in young children in 2015: a systematic review and modelling study. Lancet (2017) 390(10098):946–58. 10.1016/S0140-6736(17)30938-8 PMC5592248 28689664

[89] Céspedes PF Rey-Jurado E Espinoza JA Rivera CA Canedo-Marroquín G Bueno SM A single, low dose of a cGMP recombinant BCG vaccine elicits protective T cell immunity against the human respiratory syncytial virus infection and prevents lung pathology in mice. Vaccine (2017) 35(5):757–66. 10.1016/j.vaccine.2016.12.048 28065474

[90] Abarca K Rey-Jurado E Muñoz-Durango N Vázquez Y Soto JA Gálvez NMS Safety and immunogenicity evaluation of recombinant BCG vaccine against respiratory syncytial virus in a randomized, double-blind, placebo-controlled phase I clinical trial. EClinicalMedicine (2020) 27:100517. 10.1016/j.eclinm.2020.100517 33073219 PMC7548429

[91] Cautivo KM Bueno SM Cortes CM Wozniak A Riedel CA Kalergis AM Efficient Lung Recruitment of Respiratory Syncytial Virus-Specific Th1 Cells Induced by Recombinant Bacillus Calmette-Guérin Promotes Virus Clearance and Protects from Infection. J Immunol (2010) 185(12):7633–45. 10.4049/jimmunol.0903452 21084664

[92] Rey-Jurado E Soto J Gálvez N Kalergis AM A safe and efficient BCG vectored vaccine to prevent the disease caused by the human Respiratory Syncytial Virus. Hum Vaccin Immunother (2017) 13(9):2092–7. 10.1080/21645515.2017.1334026 PMC5612508 28598702

[93] Mazur NI Higgins D Nunes MC Melero JA Langedijk AC Horsley N The respiratory syncytial virus vaccine landscape: lessons from the graveyard and promising candidates. Lancet Infect Dis (2018) 18(10):e295–311. 10.1016/S1473-3099(18)30292-5 29914800

[94] Andrade CA Pacheco GA Nicolas MSG Soto JA Bueno SM Kalergis AM Pathogenesis and Resolution of hRSV and hMPV Infections. Viruses (2020) 12:637. 10.3390/v12060637 PMC7354512 32545470

[95] Van Den Hoogen BG De Jong JC Groen J Kuiken T De Groot R Fouchier RAM A newly discovered human pneumovirus isolated from young children with respiratory tract disease. Nat Med (2001) 7(6):719–24. 10.1038/89098 PMC7095854 11385510

[96] Yi L Zou L Peng J Yu J Song Y Liang L Epidemiology, evolution and transmission of human metapneumovirus in Guangzhou China, 2013–2017. Sci Rep (2019) 9(1):1–9. 10.1038/s41598-019-50340-8 31575919 PMC6773679

[97] Zhu R Guo C Zhao L Deng J Wang F Sun Y Epidemiological and genetic characteristics of human metapneumovirus in pediatric patients across six consecutive seasons in Beijing, China. Int J Infect Dis (2020) 91:137–42. 10.1016/j.ijid.2019.11.012 31821893

[98] Palavecino CE Céspedes PF Gómez RS Kalergis AM Bueno SM Immunization with a Recombinant Bacillus Calmette–Guérin Strain Confers Protective Th1 Immunity against the Human Metapneumovirus. J Immunol (2014) 192(1):214–23. 10.4049/jimmunol.1300118 24319265

[99] Coccia EM Battistini A Early IFN type I response: Learning from microbial evasion strategies. Semin Immunol (2015) 27(January):85–101. 10.1016/j.smim.2015.03.005 25869307 PMC7129383

[100] MontoAS Epidemiology of Respiratory Infection. Am J Med (2002) 112(6A):4S–12S. 10.1016/S0002-9343(01)01058-0 11955454

[101] Dockrell HM Smith SG What have we learnt about BCG vaccination in the last 20 years? Front Immunol (2017) 8(SEP):1–10. 10.3389/fimmu.2017.01134 28955344 PMC5601272

[102] O’Neill LAJ Netea MG BCG-induced trained immunity: can it offer protection against COVID-19? Nat Rev Immunol (2020) 20(6):335–7. 10.1038/s41577-020-0337-y PMC7212510 32393823

[103] Mantovani A Netea MG Trained Innate Immunity, Epigenetics, and Covid-19. N Engl J Med (2020) 383(11):1078–80. 10.1056/NEJMcibr2011679 32905684

[104] Netea MG Giamarellos-Bourboulis EJ Domínguez-Andrés J Curtis N van CR van de Veerdonk FL Trained immunity: a tool for reducing susceptibility and severity of SARS-CoV2 infection. Cell Press (2020) 181(5):P969-77. 10.1016/j.cell.2020.04.042 PMC7196902 32437659

[105] Gupta PK New disease old vaccine: Is recombinant BCG vaccine an answer for COVID-19? Cell Immunol (2020) 356(July):104187. 10.1016/j.cellimm.2020.104187 32745670 PMC7386780

[106] Kamat S Kumari M BCG Against SARS-CoV-2: Second Youth of an Old Age Vaccine? Front Pharmacol (2020) 11(July):1–6. 10.3389/fphar.2020.01050 32754036 PMC7381314

[107] Ebina-Shibuya R Horita N Namkoong H Kaneko T National policies for paediatric universal BCG vaccination were associated with decreased mortality due to COVID-19. Respirology (2020) 25(8):898–9. 10.1111/resp.13885 PMC7323121 32558034

[108] Yitbarek K Abraham G Girma T Tilahun T Woldie M The effect of Bacillus Calmette–Guérin (BCG) vaccination in preventing sever infectious respiratory diseases other than TB: Implications for the COVID-19 pandemic. Vaccine (2020) 38(41):6374–80. 10.1016/j.vaccine.2020.08.018 PMC7416741 32798142

[109] Nieuwenhuizen NE Kulkarni PS Shaligram U Cotton MF Rentsch CA Eisele B The recombinant bacille Calmette-Guérin vaccine VPM1002: Ready for clinical efficacy testing. Front Immunol (2017) 8(SEP):1–9. 10.3389/fimmu.2017.01147 28974949 PMC5610719

[110] Murray RA Mansoor N Harbacheuski R Soler J Davids V Soares A Bacillus Calmette Guerin Vaccination of Human Newborns Induces a Specific, Functional CD8+ T Cell Response. J Immunol (2014) 177(8):5647–51. 10.4049/jimmunol.177.8.5647 17015753

[111] Hanekom WA The immune response to BCG vaccination of newborns. Ann New York Acad Sci (2005) 1062:69–78. 10.1196/annals.1358.010 16461790

[112] Beyazova U Rota S Cevheroǧlu C Karsligil T Humoral immune response in infants after BCG vaccination. Tuber Lung Dis (1995) 76(3):248–53. 10.1016/S0962-8479(05)80013-9 7548909

[113] Cirauqui C Benito-Villalvilla C Sánchez-Ramón S Sirvent S Diez-Rivero CM Conejero L Human dendritic cells activated with MV130 induce Th1, Th17 and IL-10 responses via RIPK2 and MyD88 signalling pathways. Eur J Immunol (2018) 48(1):180–93. 10.1002/eji.201747024 PMC5813220 28799230

[114] Ehreth J The global value of vaccination. PharmacoEconomics Outcomes News (2003) 21:596–600. 10.1016/S0264-410X(02)00623-0

